# Dynamics of soil properties and bacterial community structure by mulched fertigation system in semi-arid area of Northeast China

**DOI:** 10.7717/peerj.14044

**Published:** 2022-09-22

**Authors:** Ling Wang, Meng Wang, Qian Li, Jinjing Zhang, Cuilan Li, Yuhan Yuan, Pan Tan, Hang Liu

**Affiliations:** 1 Key Laboratory of Soil Resource Sustainable Utilization for Commodity Grain Bases of Jilin Province, College of Resource and Environmental Science, Jilin Agricultural University, Changchun, Jilin, China; 2 Institute of Agricultural Environment and Resources Research, Jilin Academy of Agricultural Sciences, Changchun, China

**Keywords:** Bacterial community, Illumina MiSeq sequencing, Mulched fertigation, Soil properties

## Abstract

The agricultural irrigation and fertigation systems have a non-negligible impact on the soil microenvironment in arid and semi-arid areas. Therefore, studying the processes and changes of soil microenvironment under different plastic mulch drip irrigation systems can reveal the “soil-microbe” mechanism and provide a theoretical support for the optimal irrigation and nutrition management of maize in the semi-arid area of Northeast China. Three treatments were used for this study in the semi-arid area of northeast China, namely; mulched fertigation system (MF), drip irrigation system (DI), and farmers’ practices system (FP). We used high-throughput sequencing to study the soil bacterial community structure targeting the 16S rRNA gene. The agricultural irrigation and fertigation systems significantly affected soil properties. MF significantly increased bacterial abundance and bacterial diversity and richness. Moreover, MF and DI markedly increased some relative abundance of beneficial bacterial. The bacterial network in MF was more conducive to the health and stability of the agroecosystem and the relationships among species in MF bacterial network were more complex. The agricultural irrigation and fertigation systems had indirect effects on community composition and bacterial diversity through soil organic carbon (SOC), ammonium nitrogen (${\mathrm{NH}}_{4}^{+}$-N), nitrate nitrogen (${\mathrm{NO}}_{3}^{-}$-N), pH, moisture, ${\mathrm{NH}}_{4}^{+}$-N and ${\mathrm{NO}}_{3}^{-}$-N had indirect effects on yield through bacterial community composition, bacterial diversity and bacterial abundance. These findings suggested that MF was the most effective treatment to improve soil bacterial abundance and diversity, and stabilize the functional quality of soil biological processes.

## Introduction

The semi-arid area in China is influenced by natural environmental factors like drought and soil nutrient depletion; due to the rainfall and crop water requirements mismatch, soil water supply is often one of the important limiting factor of crop productivity. However, the cultivation of maize is known to be largely dependent on water and soil nutrients. The traditional methods for managing water and fertilizer, like flooding irrigation and the single basal fertilizer application, are known to result in lower nutrient use efficiency compared with high nutrient use efficiency and severely restrict crop yield (Ju et al., 2007; Vitousek et al., 2009). Several studies had been carried out to develop and promote new agricultural technologies that will enhance nutrient use efficiency and also improve crop yield. In recent times, the mulched fertigation (MF) system has appeared to be the most promising technology, which provides a technical implementation path for achieving high yields and high nutrient/water use efficiencies (Qin et al., 2016).

The MF system combines plastic film mulch and fertigation technology, where the plastic film can increase soil temperature through the conductive transfer of solar energy during the day. Moreover, the plastic mulch reduce evaporation because of their water-impermeable nature. This process makes the soil environment conducive for microbial activities thereby improving decomposition and the release of nutrients into the soil (Gan et al., 2013). The MF system significantly improves water and nutrient use efficiency, promote crop production, improve product quality, and improve soil microbial environment and soil nutrient (De Pascale & Barbieri, 1995; Hou et al., 2014). Additionally, the MF system can effectively control fertilizer and water applications at any time during the growing season according to the nutritional requirements of crops, and effectively regulate soil evaporation, reduce the risk of soil degradation and saline-alkali hazards (Ajdary et al., 2007; Martins et al., 2013; Yang et al., 2017).

Soil bacteria are important in agricultural ecosystems and soil function (Heijden & Wagg, 2013). Bacterial abundance and community structure have sensitive responses to changes in environmental conditions. They play an important role in element cycling, organic matter degradation, and nutrient turnover, and directly or indirectly affect the growth of crops. Bacteria perform important functions in the soil, decomposing organic residues from enzymes secreted in the soil. Decomposers are bacteria that consume simple sugars and simple carbon compounds, such as root exudates and fresh plant litter. Bacteria mutualists form partnerships with plants including the nitrogen-fixing bacteria. Bacteria can also become pathogens to plants. Lithotrophs or chemoautotrophs bacteria obtain energy from compounds of nitrogen, sulfur, iron, or hydrogen instead of from carbon compounds. Some of these species are important to nitrogen cycling and degradation of pollutants (Hoorman, 2011). The direction of modern agriculture is now focused on appropriate agricultural management practices that to develop the potential of soil microbiology. It is well known that the soil bacteria diversity and community composition are primarily influenced by environmental factors and agricultural management practices (Van Elsas, Garbeva & Salles, 2002). Especially in arid and semi-arid areas, the irrigation systems have an impact on soil microenvironment. The different irrigation systems alter soil water and heat conditions and have a direct or indirect impact on bacterial abundance and diversity. The irrigation system changed the soil water content and pH, so it also had a certain impact on the relative abundance of the dominant groups of bacteria in the soil, significantly changing the relative abundance of Actinobacteria, Bacteroidea and Saccharibacteria (Lauber et al., 2008). The depth of the subsurface drip irrigation tape was an important factor affecting soil microbes (Fu et al., 2021). Moreover, the plant biomass, together with the soil water content, was also found to affect the soil microbial functional diversity (Liu et al., 2010). The variation of soil moisture, can directly or indirectly affect soil bacterial abundance, and community structure. Furthermore, the soil moisture distribution in different buried depths of drip tape can also differentiate root growth and therefore regulates and controls the interaction between root growth and the soil bacteria community (Wang, Li & Niu, 2020).

Soil microbial community is implicated in numerous processes such as the biochemical transformation of carbon and nitrogen and the development of soil physical structure, sustainable crop productivity, and soil ecosystem health. Being a source and sink of soil nutrients, structural alterations in the microbial communities also influence the rate of nitrogen fixation, nitrification, and de-nitrification, processes of nutrient cycling, and thus crop production (Philippot et al., 2011). Soil microbial community can react promptly to the subtle alterations in soil conditions. As a result, soil microbes serve as a key ecological indicator for monitoring tiny modifications in the soil microenvironment brought by agronomic management practices such as mulching and hence could have huge impacts on ecosystem functioning and dynamics (Shen, Chen & Li, 2016).

Therefore, studying the process of soil microenvironment changes under different plastic mulch drip irrigation systems can provide a better understanding to determine the best irrigation and fertilization management method, so as to optimize the crops yield. Some investigations have reported MF system had a significant positive impact on the characteristics of soil water and heat migration, crop yield, nutrient efficiency and water productivity (Liu et al., 2017; Wang et al., 2019; Wang et al., 2017b), while ignoring their effects on the soil microenvironment and soil microbial community structure. Oved et al. (2001) reported that wastewater effluent and fertilizer-amended water (FAW) irrigation systems had a significant influence on the ammonia-oxidation bacteria community in the vineyard soil, *Nitrosospira* and *Nitrosomonas* were dominant in soils irrigated with FAW, and effluent-irrigated soils. Zhang et al. (2015) found that nitrogen application and increased soil moisture were beneficial to the maintenance of bacterial diversity and ecological functions. Wang et al. (2017a) manifested that the soil alkaline phosphatase activity was most distinct to the change in irrigation systems. However, the research on the response of soil bacteria to the soil environmental change of drip irrigation with plastic film mulching was not comprehensive, and the research methods were mostly traditional culture methods, which could not reflect the soil microbial information deeply. the comprehensive research on the changes of soil bacterial community structure has yet to be addressed under FP (farmers’ practices system), DI (drip irrigation system), and MF systems. The reproduction of soil microorganisms is fundamentally affected by abiotic factors. The water resources are becoming increasingly scarce, especially in arid and semi-arid areas, water has become a more critical constraint factor. Irrigation systems have a non-negligible impact on soil microenvironment changes. Therefore, studying the changes of soil bacteria under different irrigation systems can reveal the interaction mechanism between soil and bacteria, and provide a theoretical basis for the rational allocation of irrigation technology and management systems for crops. The purpose of this study was to evaluate the changes in soil chemical properties, bacterial abundance, and community structure variation under FP, DI and MF systems using quantitative real-time PCR (qPCR) and high-throughput sequencing methods. We hypothesized that MF will enhance the bacterial microbial activities and increase the bacterial microbial abundance due to an increased soil temperature and moisture.

## Materials and Methods

### Study site description and soil sample collection

This study was conducted in Minle Village (45°26′N, 125°88′E), Jilin Province, Northeast China. The soil at the experiment site is chernozem sandy loam, with 17.68 g kg^−1^ soil organic matter, 105.83 mg kg^−1^ available N, 12.79 mg kg^−1^ available P, 104.52 mg kg^−1^ available K and pH 7.6. The long-term field experiment was established in 2015 and had since been subjected to continuous maize cropping. Maize was sown in early May and harvested in early October. The maize cultivar used was FuMin-985, planting density was 70,000 plants ha^−1^, and sowing depth was two cm. The area size of each treatment was 300 m^2^ (15 by 20 m). After the maize entered the maturity period, the maize harvester was used for harvesting. After harvest, used a 1JHY-200 straw chopper to crush maize straw (length < 10 cm), and evenly scatter it in the field. The 1LYFT-450 fence type hydraulic-overturning plough was used to plough the straw into the soil (the power was more than 140 horsepower, and the ploughing depth was 30–35 cm), and turn the straw to a depth of 20–30 cm soil layer. The combined land leveler was used to spin and rake to reach the sowing state. When the soil temperature of five cm was stable and passed 8 °C, adopted wide and narrow rows for planting. After heavy suppression (the suppression strength was 400–800 g/cm^2^), we used the 2BJHM-2 multifunctional water-saving mulching planter to complete fertilization, sowing, drip irrigation pipes, film mulching, and soil covering at the same time. After the experiment set up, the crop type, cropping density, fertilizer application rate, crop straw management are consistent.

The experiment included three treatments: mulched fertigation system (MF), drip irrigation system (DI), and farmers’ practices system (FP). The three treatments were arranged in a completely random block design with three repetitions. Under treatment MF, the non-degradable plastic film that a conventional white polyethylene (PE) plastic mulch for maize with a width of 110 cm and a thickness of 0.008 mm was selected, and the amount was 66.45 kg ha^−1^. Maize was sown in broad-narrow ridges pattern, with broad and narrow rows’ spaces of 80 and 40 cm, respectively, both ridges were covered with the plastic film. Each plot was supplied with an independent unit of the gravity drip irrigation system, which was a dripline placed in the middle of each narrow row, and the emitter spacing was 30 cm. A fertilizer tank was installed upstream of the pressure regulating valve to apply fertilizer. The nominal flow rate of the dripper was 2.0 L h. In treatment DI, the shallow-buried drip irrigation system was applied with no plastic mulch. In treatment FP, the fertilizer and water management were controlled by local farmers’ management practices, without plastic mulch and irrigation by flooding with water pipe. The irrigation amount of the three treatments was consistent. The total irrigation amount of three treatments in maize growth period were 220 mm, of which the irrigation amount at before sowing (BS) and eighth leaf stage were 20 mm, and the irrigation amount in twelfth leaf, tasseling stage (VT), and milk stage (R3) were 60 mm. Each treatment was irrigated at the same time, and the same irrigation amount was controlled by water meter. The test water source was groundwater. Each irrigation system received equal amounts of 210 kg N ha^−1^, 90 kg P_2_O_5_ ha^−1^, and 90 kg K_2_O ha^−1^. Under MF and DI systems, the total N was split into five applications of 30, 30, 20, 10, and 10% BS, eighth leaf, twelfth leaf, VT, and R3, respectively. The total P_2_O_5_ and K_2_O were divided into five applications of 50, 20, 15, 10, and 5% at the five growth stages. In addition, urea, triple superphosphate, and potassium chloride were dissolved in the irrigation water and applied by drip fertigation. However, under treatment FP the total N was applied once (100%) before sowing while the total P_2_O_5_ and K_2_O were applied with the same proportion as DI and FP at the five growth stages.

Soil samples for this study were collected at BS (5.5), sixth leaf (V6) (6.26), VT (7.24) and harvest stages (R6) (9.27) in 2019. Soil samples were taken on the 7th day after irrigation fertilization at BS and VT. Under each treatment, eradicated the vegetation and weeds on the soil surface carefully, five soil samples were randomly collected from the tillage layers (0–20 cm) with a soil auger and mixed into them to form a composite sample. Each composite sample was about 1 kg. Each treatment was composed of four replicated composite samples. The soil samples were placed into respective sterile bags in an icebox and carried back to the laboratory. The samples were sieved with a 2-mm mesh to remove roots and stones and a part of the soil samples were stored at −80 °C for soil DNA extraction. Other soil samples were air-dried at room temperature to determine soil physicochemical properties.

### Soil physicochemical properties and maize yield analysis

The soil physicochemical properties were determined according to the methods of Lu (2000). The soil pH was measured by the Potentiometric method. Weigh 5 g of air dried soil, add 12.5 ml of distilled water according to the ratio of water: soil = 2.5:1, and then place in the room temperature oscillator to vibrate for 1 h (180 RPM). After standing for 30 min, measure it with a pH meter. The soil moisture content was mass water content of the soil, taking about 25 g of fresh soil and putting it into the oven drying at 105 °C for 6–8 h, and calculating the soil moisture content through the calculation formula (water weight *100/ dry weight). Soil organic carbon (SOC) was measured with an air-dried soil sample sieved through a 0.25 mm sieve and measured with an elemental analyzer (VarioEL III; Elementar, Hanau, Germany). Soil ammonium nitrogen (${\mathrm{NH}}_{4}^{+}$-N) and nitrate nitrogen (${\mathrm{NO}}_{3}^{-}$-N) were measured with a continuous flow analyzer (AA3; Seal, Germany): take 10 g of fresh soil sample passing through a two mm sieve, add 100 ml of 2 M KCl solution, shake the table for 1 h, and filtered with filter paper. Soil available nitrogen (AN) was measured with alkali hydrolysis and diffusion method: Take 2.00 g of air dried soil and put it in the outer chamber of the diffusion dish to make the soil evenly spread. Add 0.2 g of ferrous sulfate powder and 2 ml of H_3_BO_3_ indicator solution in the inner chamber of the diffusion dish. Quickly add 10 ml of NaOH solution, and then put it into a 40 °C incubator. After alkali hydrolysis and diffusion for 24 h, remove it. Titrate NH_3_ in the absorption solution in the inner chamber with a sulfuric acid standard solution. The solution changes from blue to reddish as the titration end point. Soil available phosphorus (AP) was measured with the molybdenum blue method. Weigh 5.00 g of air-dried soil, add a small spoonful of phosphorus-free activated carbon, 100 ml of 0.5 mol/l NaHCO_3_ extraction solution, shake for 30 min, remove for filtration, suck 10 ml of the filtrate, add 5 ml of molybdenum antimony anti developer, and compare the color on the spectrophotometer after 30 min. Available potassium (AK) was measured with flame photometry (FP640, INASA, China). Weigh 5.00 g of air-dried soil into a plastic bottle, add 50 ml of 1 m ammonium acetate solution, shake for 30 min, and remove it for filtration. The filtrate was measured directly on the flame photometer. Three points were randomly selected in the harvest period, the harvest area of each yield measuring plot was 30 m^2^. The maize aboveground part of 30 m^2^ was harvested, threshed, and the seeds were dried to calculate the maize grain yield (water content 14%).

### Soil DNA extraction and quantitative real-time PCR (Q-PCR)

A total of 0.5 g fresh soil sample was weighed, using PowerSoil DNA Isolation Kit (MoBio, Carlsbad, CA, USA) and the total soil DNA was extracted according to the instructions. DNA quality and concentration were detected by NanoDrop spectrophotometer 2000 (Thermo Scientific, Waltham, MA, USA) and checked in 1% agarose gel electrophoresis.

The bacterial 16S rRNA gene was amplified using the primer set F515 (5′-GTG CCA GCM GCC GCG G-3′) and R907 (5′-CCG TCA ATT CMT TTR AGT TT-3′) (Yusoff et al., 2013). Q-PCR was quantified in an ABI 7500 Real-Time PCR System (Appiled Biosystems, Carlsbad, CA, USA) in triplicate. The PCR reactions contained 15 µL of AceQ® SYBR Green qPCR Master Mix (2X), 2 µL of Mg^2+^ (25 mM), 0.5 µL of (10 µM) each forward and reverse primers, 2.0 µL of template DNA, 0.5 µL of dyestuff and 9.5 µL of sterile double-distilled water (ddH_2_O). The PCR reaction procedure was performed as follows: initial activation at 95 °C for 3 min, 30 cycles of denaturation at 94 °C for 30 s, annealing at 60 °C for 30 s, and elongation at 72 °C for 30 s. The PCR products were checked in a 2% agarose gel and purified using AxyPrep DNA Gel Extraction Kit (Axygen Biosciences, Union City, CA, USA) and quantified by QuantiFluor™ _ST (Promega, WI, USA) according to the manufacturer’s instructions.

### 16S rRNA gene sequencing and bioinformatics analysis

The bacterial 16S rRNA was amplified using the primer set F515/R907 in a thermocycler PCR system (GeneAmp 9700; ABI, Foster, CA, USA) (Yusoff et al., 2013). The PCR reactions were performed in triplicate using a 20 µL mixture containing 4 µL of 5× FastPfu Buffer, 2µL of 2.5 mM dNTPs, 0.8 µL of each primer (5 µM), 0.4 µL of FastPfu Polymerase and 10 ng of template DNA. The amplification was performed at 95 °C for 5 min, followed by 27 cycles at 95 °C for 30 s, at 55 °C for 30 s, and at 72 °C for 45 s, and a final extension at 72 °C for 10 min, 10 °C until halted by the user. The PCR products were purified (AxyPrep PCR Clean-up Kit, Axygen Biosciences, CA, USA) before performing agarose gel electrophoresis and sequenced on an Illumina MiSeq platform at Shanghai Biozeron Biological Technology Co. Ltd.

The raw sequence data were analyzed using QIIME software (http://qiime.org/tutorials/tutorial.html) and merged by FLASH. An average quality score < 20 and < 200 bp in length were removed. The Uchime algorithm was used to eliminate chimera from noisy sequences (Edgar et al., 2011). The high-quality sequences were clustered into operational taxonomic units (OTUs) using the UPARSE pipeline (version 10, http://drive5.com/uparse/) at 97% similarity level (Edgar, 2013). The OTU representative sequence was aligned using the Python nearest alignment space termination (PyNAST) algorithm (Desantis et al., 2006). A phylogenetic tree was built using FastTree (Price, Dehal & Arkin, 2009). Taxonomic information was analyzed by Silva (Release132 http://www.arb-silva.de) against the 16S rRNA database using confidence threshold of 0.80 (Quast et al., 2013). In order to analyze the microbial communities at the same sequencing depth, the lowest sequencing number of 28,602 sequences for bacterial 16S rRNA gene were randomly selected per sample. The raw sequences were deposited into NCBI under the accession number SRP365802.

### Statistical analysis

Significant differences in soil chemical properties, bacterial abundance, and alpha diversity among treatments were determined by the *F* test in one-way ANOVA, and multiple comparisons of means (*P* < 0.05) were conducted with a Fisher’s protected least significant difference (LSD) using SPSS software version 20. The student’s *t*-test was used to assess differences in the relative abundances at phylum and genera levels with 95% confidence intervals in STAMP 2.1.3 (http://kiwi.cs.dal.ca/Software/STAMP) (Parks et al., 2014). A heatmap was performed using the pheatmap package in the R software (version 3.5.0) (R Core Team, 2010). The log of the response ratio (LRR) was computed as follows: LRR = ln ($\overline{X}1$/ $\overline{X}2$) (Hedges, Gurevitch & Curtis, 1999), where X1 was the relative abundance of genera in MF and DI, X2 was that in FP. LRR was performed using the readxl package in the R software (version 3.5.0) (R Core Team, 2010). The Shannon index, which indicates community diversity within the sample, was determined as follows:

\begin{eqnarray*}\text{Shannon}=-\sum _{i=1}^{\mathrm{Sobs}} \frac{ni}{N} \mathrm{ln} \frac{ni}{N} \end{eqnarray*}

where Sobs is the number of observed OTUs, n1 is the number of individuals in the OTU, and Nis is the total number of individuals in the community (Jurasinski, Retzer & Beierkuhnlein, 2009). The Chao1 index (the estimated number of OTUs), which indicates species richness, was calculated using the following equation:

\begin{eqnarray*}\text{Chao}1=\text{Sobs}+ \frac{n1(n1-1)}{2(n2+1)} \end{eqnarray*}

where Sobs is the observed number of OTUs, n1 is the number of OTUs with only one sequence, and n2 is the number of OTUs with only two sequences (Liu et al., 2014). Principal coordinate analysis (PCoA) and Adonis based on the Bray-Curtis distance were performed using the vegan package in the R software (version 3.5.0) (R Core Team, 2010). Linear discriminant analysis (LDA) effect size (LEfSe) was drawn using the Huttenhower Galaxy web at the LEfSe algorithm (http://huttenhower.sph.harvard.edu/galaxy/). The LDA was considered to be an important contributor with an LDA score ≥ 2.0. The Pearson correlation analysis between soil bacterial communities and soil physicochemical properties was performed in the PerformanceAnalytics package in the R environment (version 3.5.0) (R Core Team, 2010). Structural Equation Modeling (SEM) was performed using AMOS (version 24). Model adequacy was determined by *χ*^2^ tests (*P* > 0.05), goodness-of-fit index (GFI > 0.9), Akaike Information Criteria (AIC), and root square mean errors of approximation (RMSEA < 0.08). Co-occurrence network visualization was performed in Gephi software (0.9.2) with the robust Spearman correlations coefficient *ρ* > 0.7 and *P* < 0.05.

## Results

### Soil physicochemical properties and maize yield

**Figure 1:**
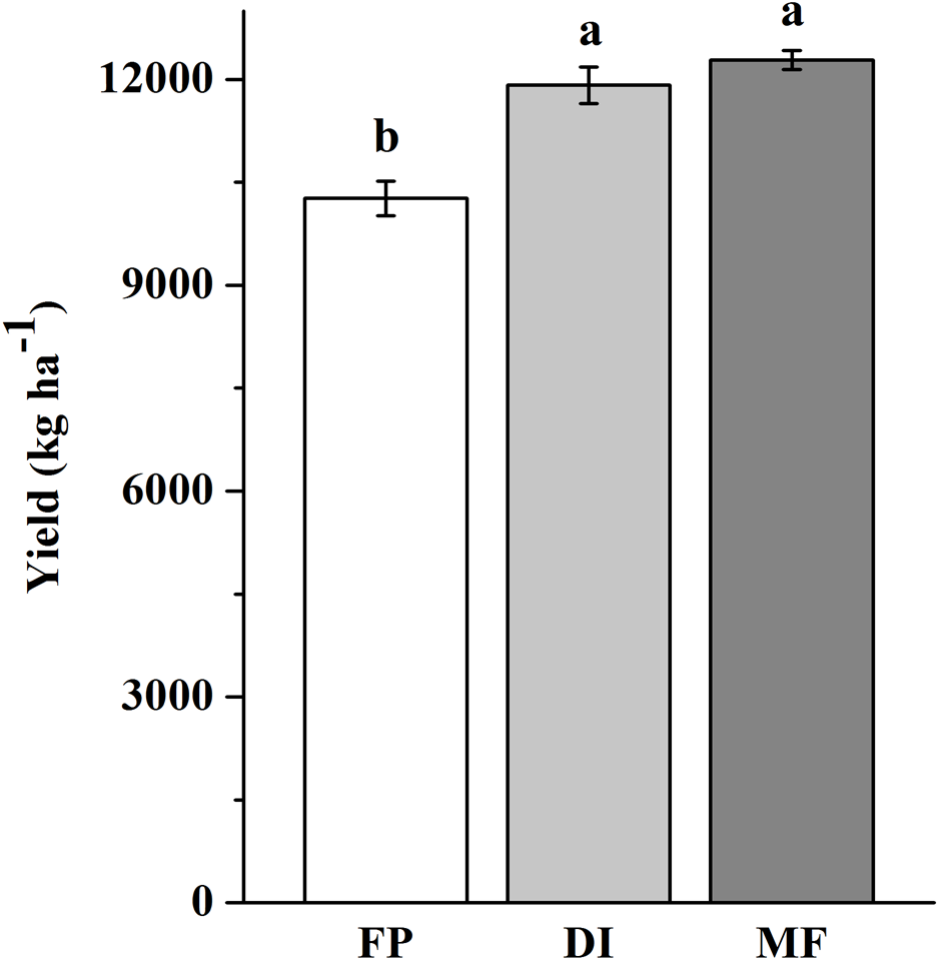
Maize yield in three agricultural cropping patterns. MF, mulched fertigation system, drip irrigation under plastic mulch; DI, drip irrigation system, drip irrigation without plastic mulch; FP, farmers’ practices system.

The three agricultural irrigation and fertigation systems significantly affected the investigated soil properties (Table 1). Compared with FP, DI and MF significantly increased soil moisture at BS and R6, and decreased SOC content at four stages, decreased ${\mathrm{NH}}_{4}^{+}$-N content at VT, decreased ${\mathrm{NO}}_{3}^{-}$-N and AN contents at V6 and R6, decreased AP and AK contents at BS and VT (*P* < 0.05). The ANOVA results showed that ${\mathrm{NO}}_{3}^{-}$-N content at BS and VT, ${\mathrm{NH}}_{4}^{+}$-N content at BS and R6, and AP and AK contents at V6 significantly decreased in MF compared with FP (*P* < 0.05). Compared with FP, DI and MF increased the maize yield by 16 and 20%, respectively (Fig. 1).

**Table 1:** Soil chemical properties under three agricultural cropping patterns.

**Treatment**	**pH**	**Moisture (%)**	**SOC** ^b^ **(g/kg)**	${\mathrm{NH}}_{4}^{+}$-N (mg/kg)	${\mathrm{NO}}_{3}^{-}$-N (mg/kg)	**AN (mg/kg)**	**AP (mg/kg)**	**AK (mg/kg)**
FP_BS^a^	6.82 ± 0.01a^c^	13.62 ± 0.18b	13.92 ± 0.02a	0.96 ± 0.05a	10.77 ± 0.69ab	489.90 ± 25.90a	26.96 ± 0.97a	184.06 ± 2.01a
DI_BS	6.76 ± 0.01b	14.83 ± 0.19a	13.06 ± 0.12b	0.95 ± 0.03a	14.88 ± 3.19a	461.50 ± 7.49a	20.33 ± 0.62b	152.60 ± 8.15b
MF_BS	6.76 ± 0.01b	14.42 ± 0.31a	13.22 ± 0.08b	0.76 ± 0.08b	7.76 ± 1.07b	450.13 ± 17.10a	19.88 ± 1.52b	141.46 ± 2.72b
FP_V6	6.92 ± 0.01b	17.58 ± 0.29a	13.72 ± 0.04a	0.88 ± 0.21a	9.82 ± 1.24a	381.96 ± 10.20a	22.48 ± 0.95a	160.20 ± 9.92a
DI_V6	6.93 ± 0.01b	19.11 ± 2.22a	11.61 ± 0.02c	0.86 ± 0.03a	5.21 ± 0.67b	326.60 ± 15.80b	21.50 ± 0.98ab	157.29 ± 3.57ab
MF_V6	6.96 ± 0.01a	18.42 ± 0.10a	12.41 ± 0.09b	0.81 ± 0.10a	4.65 ± 0.85b	312.80 ± 6.97b	18.90 ± 1.73b	142.90 ± 3.52b
FP_VT	6.36 ± 0.02c	15.31 ± 0.23a	12.96 ± 0.12a	1.09 ± 0.05a	10.92 ± 0.74a	352.89 ± 14.70a	26.95 ± 1.16a	211.20 ± 11.20a
DI_VT	6.64 ± 0.03b	14.01 ± 0.37b	12.33 ± 0.05b	0.88 ± 0.03b	9.31 ± 0.60ab	347.36 ± 8.29a	21.76 ± 2.50b	159.40 ± 20.40b
MF_VT	6.89 ± 0.02a	15.74 ± 0.33a	11.41 ± 0.13c	0.78 ± 0.04b	7.94 ± 1.32b	332.14 ± 7.38a	15.51 ± 1.01c	131.80 ± 12.70b
FP_R6	7.15 ± 0.01a	10.78 ± 0.08c	15.67 ± 0.01a	0.99 ± 0.06a	10.37 ± 0.47a	398.56 ± 18.30a	26.23 ± 4.26a	163.50 ± 7.59a
DI_R6	7.04 ± 0.03b	11.52 ± 0.33b	14.30 ± 0.37b	0.93 ± 0.04a	6.23 ± 0.61b	340.44 ± 6.44b	23.37 ± 0.45a	140 ± 2.40b
MF_R6	6.86 ± 0.01c	13.73 ± 0.17a	12.85 ± 0.07c	0.79 ± 0.03b	6.88 ± 0.55b	316.91 ± 8.47b	21.23 ± 1.03a	137.80 ± 13.90b

**Notes.**

a MF, mulched fertigation system, drip irrigation under plastic mulch; DI, drip irrigation system, drip irrigation without plastic mulch; FP, farmers’ practices system; BS, before sowing stage; V6, sixth leaf stage; VT, flowering stage; R6, harvest stage. SOC, soil organic carbon.

b ${\mathrm{NH}}_{4}^{+}$-N, ammonium nitrogen; ${\mathrm{NO}}_{3}^{-}$-N, nitrate nitrogen; AN, available nitrogen; AP, available phosphorus; AK, available potassium.

c Different letters in the same column indicates a significant difference (*P* < 0.05).

### Soil bacterial abundance and bacterial community composition

**Figure 2:**
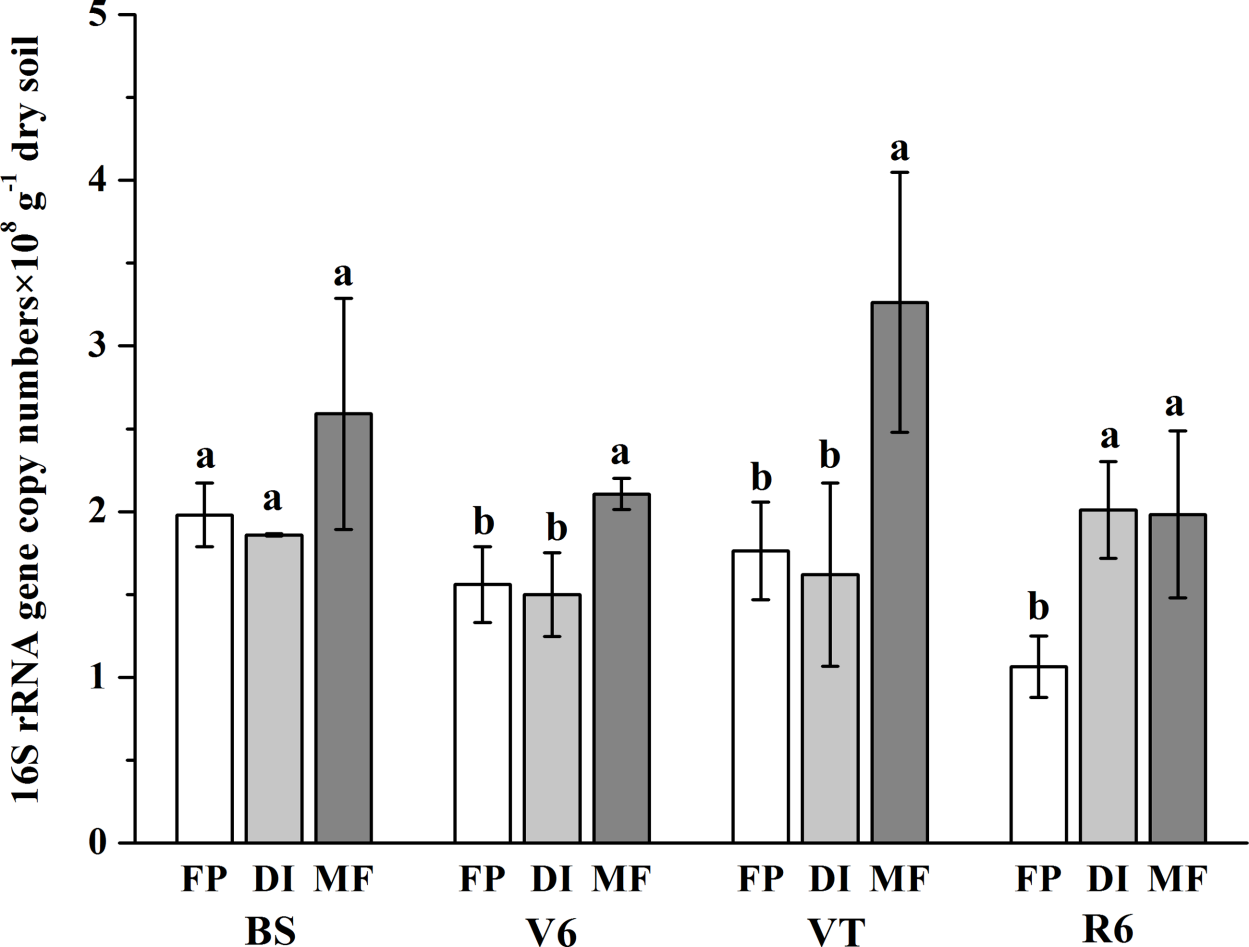
Abundance of soil bacterial 16S rRNA detected across three agricultural cropping patterns. MF, mulched fertigation system, drip irrigation under plastic mulch; DI, drip irrigation system, drip irrigation without plastic mulch; FP, farmers’ practices system; BS, before sowing stage; V6, sixth leaf stage; VT, flowering stage; R6, harvest stage.

The bacterial abundance varied from 1.07 × 10^8^ gene copy g^−1^ dry soil to 3.26 × 10^8^ gene copy g^−1^ dry soil across all samples (Fig. 2). On the whole, compared with the FP, MF significantly increased the bacterial abundance, especially at the VT stage, which was 2.49-fold higher than that of FP. The bacterial abundance was higher in DI than that in FP at the R6 stage, but no significant differences in bacterial abundance were observed between FP and DI at the BS, V6 and VT stages.

**Figure 3:**
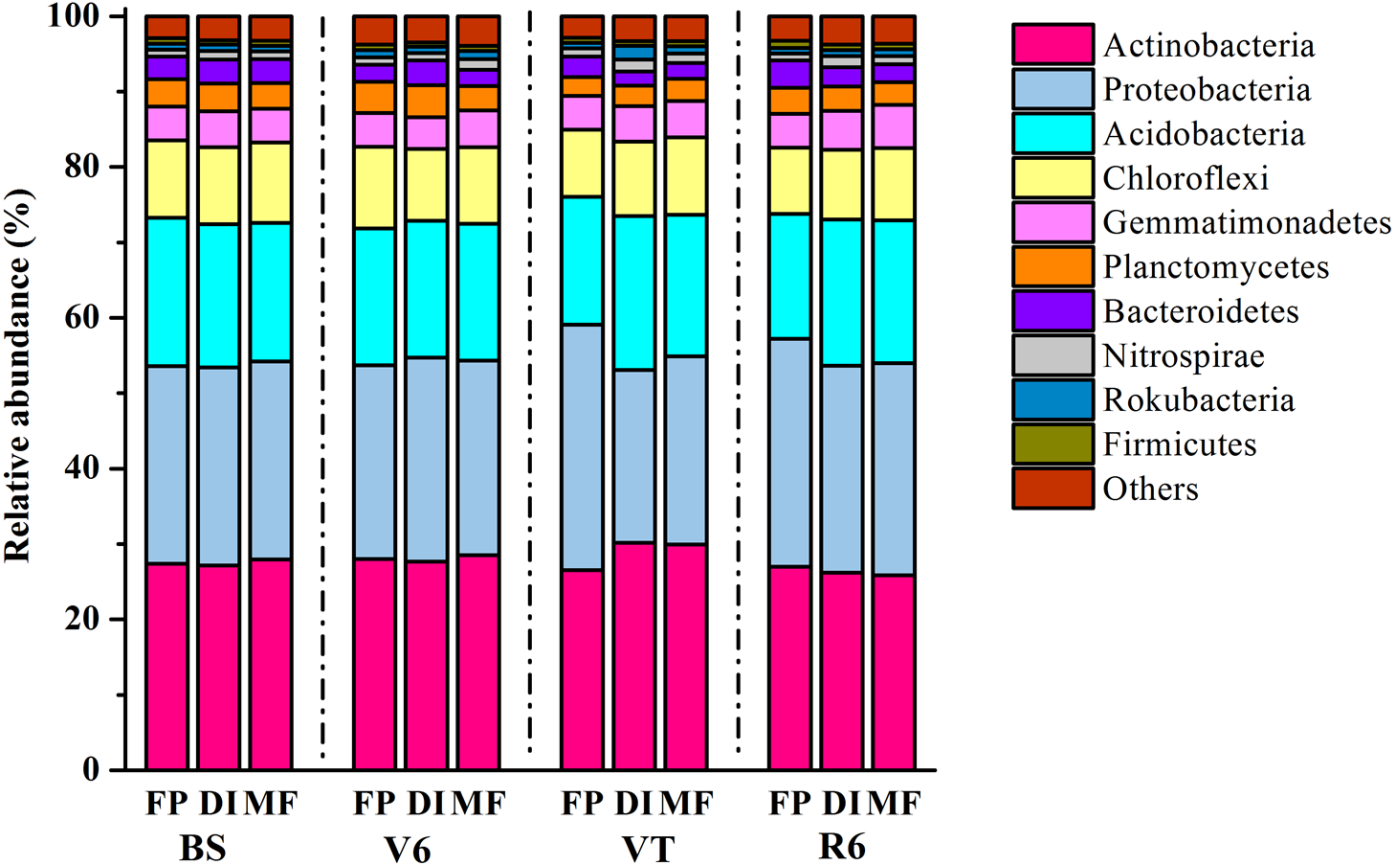
Taxonomic composition of bacterial communities at the phylum level under three agricultural cropping patterns. MF, mulched fertigation system, drip irrigation under plastic mulch; DI, drip irrigation system, drip irrigation without plastic mulch; FP, farmers’ practices system; BS, before sowing stage; V6, sixth leaf stage; VT, flowering stage; R6, harvest stage.

In total, 1,939,797 quality sequences were generated, resulting in 30,010-51,944 quality sequences per sample (Table S1). The dominant bacterial phyla across all soil samples were Actinobacteria, Proteobacteria and Acidobacteria, with relative abundance ranging from 27.16% to 30.19%, from 22.87% to 32.52% and from 16.56% to 20.46% across all samples, respectively (Fig. 3). DI and MF reduced the relative abundance of Acidobacteria compared with FP at the R6 stage (Fig. S1). Significant differences were observed in some lower abundance phyla among the three agricultural irrigation and fertigation systems. Compared with FP, the relative abundance of Nitrospirae and Armatimonadetes significantly enhanced in MF at the V6 stage and VT stage, respectively (Fig. S1). Compared with FP, DI and MF enhanced the relative abundance of Gemmatimonadetes and Omnitrophicaeota at the R6 stage and reduced the relative abundance of Chloroflexi.

**Figure 4:**
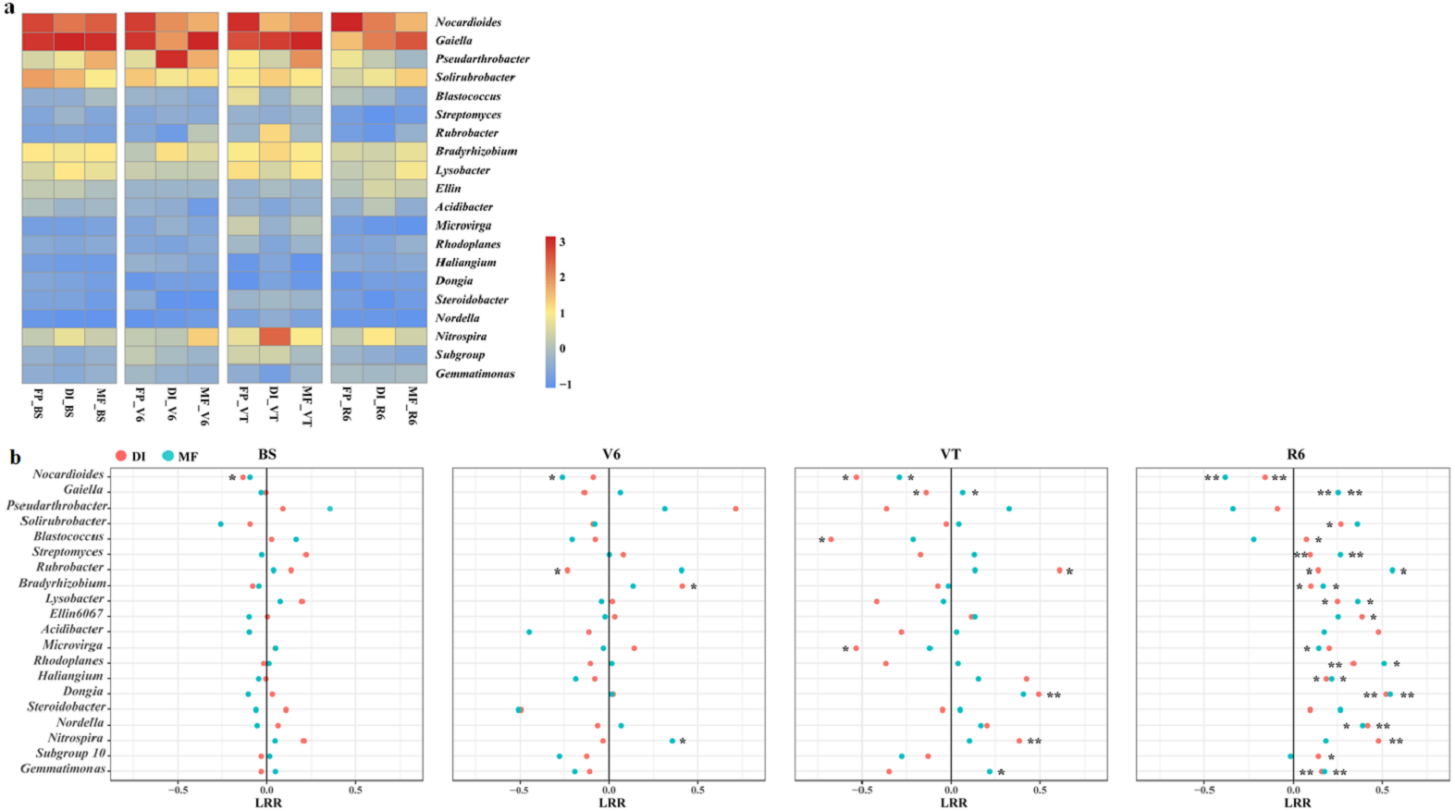
(A) Heatmap illustrating the variation of the dominant bacterial genera under three agricultural cropping patterns. The color code indicates the relative abundance of the genus, ranging from high abundance (red) to low abundance (blue). (B) Log response ratio. Significant difference in relative abundance between DI, MF and FP. *P < 0.05, **P < 0.01 by Student’s *t*-test. MF, mulched fertigation system, drip irrigation under plastic mulch; DI, drip irrigation system, drip irrigation without plastic mulch; FP, farmers’ practices system; BS, before sowing stage; V6, sixth leaf stage; VT, flowering stage; R6, harvest stage.

The heatmap of the bacterial community displayed the relative abundance of dominant bacterial genera among the three agricultural irrigation and fertigation systems (Fig. 4A), and the response of dominant genera was illustrated by calculating the LRR (Fig. 4B). The different agricultural irrigation and fertigation systems had large influences on the relative abundance of the dominant genera. The dominant genera were *Nocardioides*, *Gaiella*, *Pseudarthrobacter* and *Solirubrobacter*. DI and MF reduced the relative abundance of *Nocardioides* compared with FP. The relative abundance of *Gaiella* and *Gemmatimonas* was significantly higher in MF than that in FP at VT and R6. DI and MF resulted in an increase in the relative abundance of *Streptomyces*, *Rubrobacter*, *Bradyrhizobium*, *Lysobacter*, *Rhodoplanes*, *Haliangium*, *Dongia* and *Nordella* when compared with FP at R6. Compared with FP, MF enhanced the relative abundance of *Nitrospira* at V6 and VT while that of DI increased at VT and R6.

### Bacterial diversity and community structure

**Figure 5:**
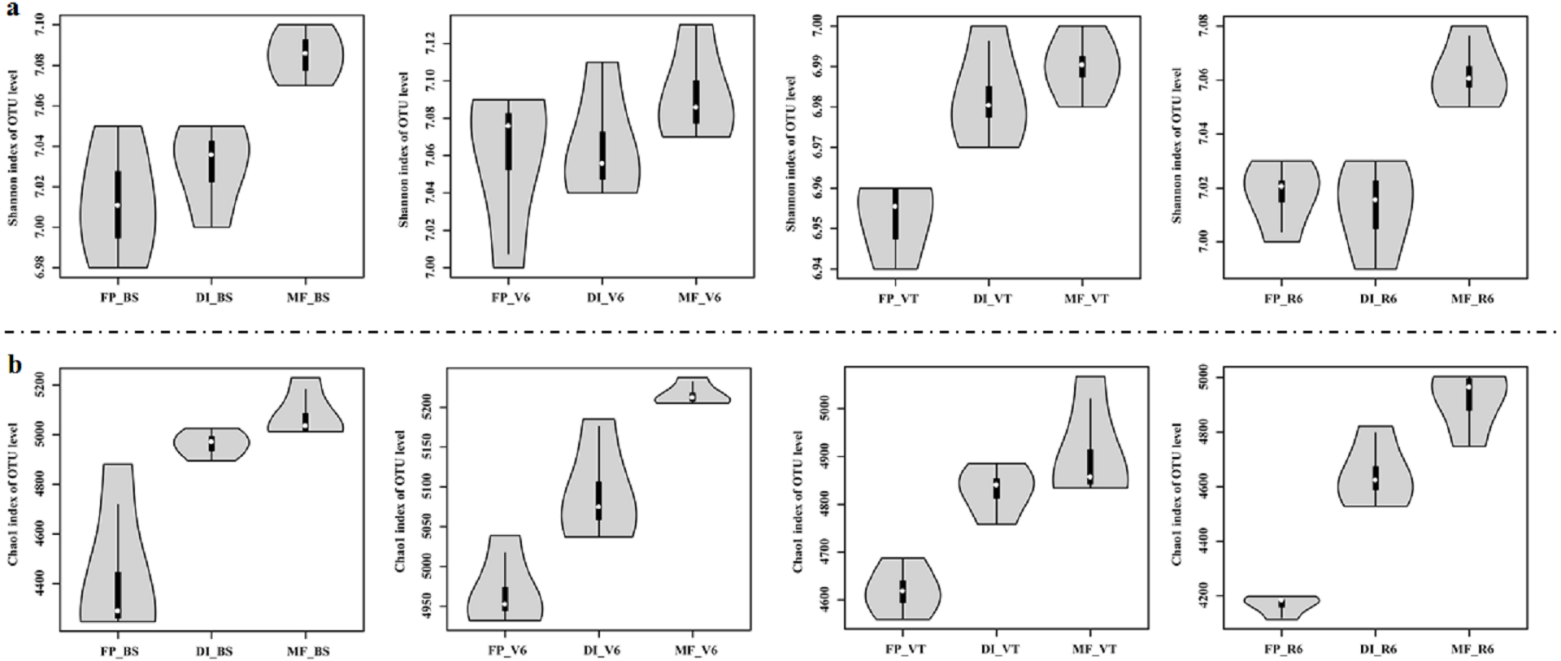
Shannon index (A) and Chao index (B) of bacterial communities at the OTU level for three agricultural cropping patterns at BS, V6, VT and R6. MF, mulched fertigation system, drip irrigation under plastic mulch; DI, drip irrigation system, drip irrigation without plastic mulch; FP, farmers’ practices system; BS, before sowing stage; V6, sixth leaf stage; VT, flowering stage; R6, harvest stage.

MF had the highest Shannon index compared with FP and DI at BS, VT and R6 (Fig. 5A). Chao1 index was significantly higher in DI and MF than that in FP. at BS, V6, VT and R6 (Fig. 5B).

**Figure 6:**
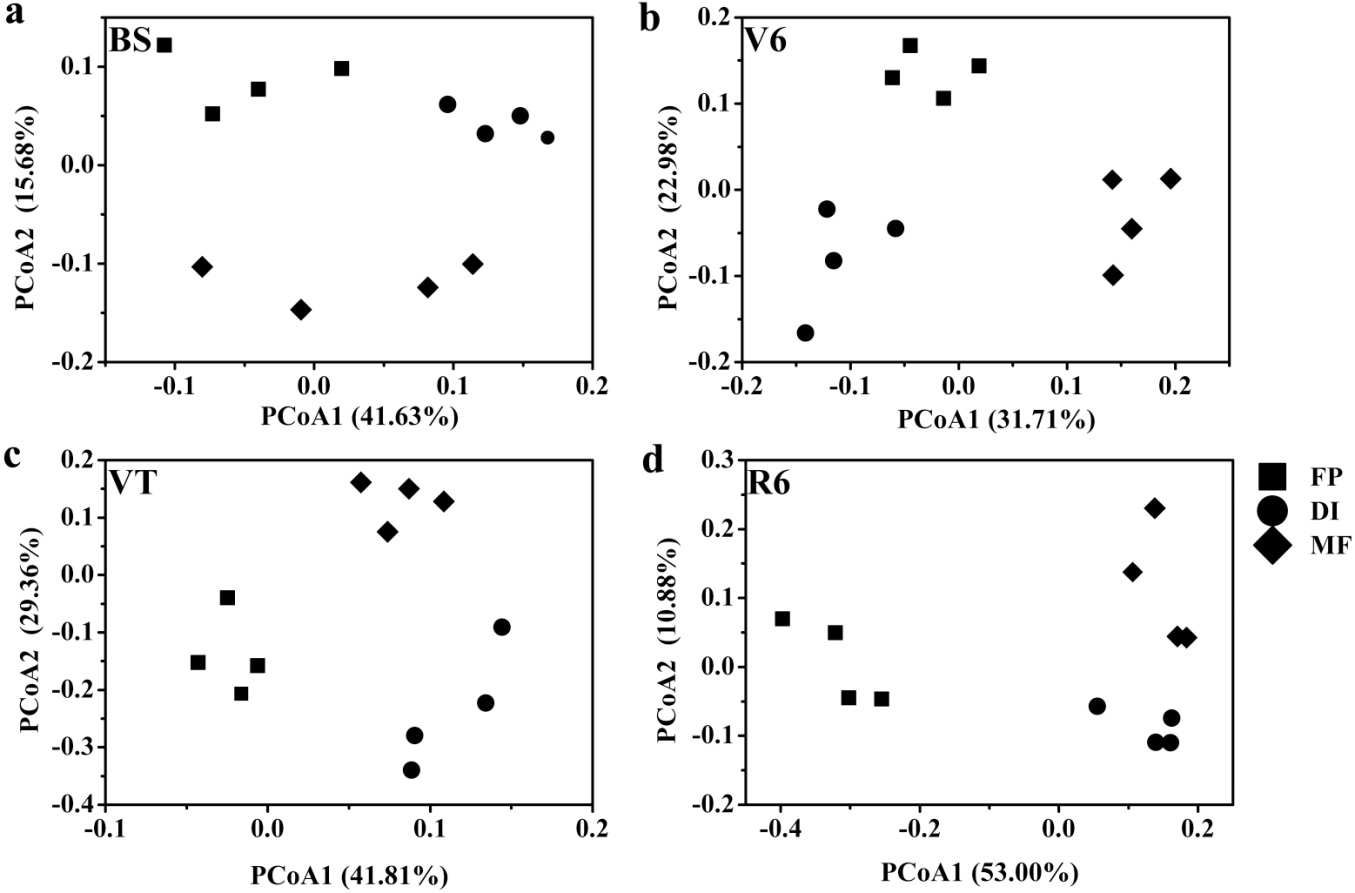
Principal coordinates analysis (PCoA) of bacterial communities based on OTUs level with Bray-Curtis distance under three agricultural cropping patterns at BS (A), V6 (B), VT (C) and R6 (D). MF, mulched fertigation system, drip irrigation under plastic mulch; DI, drip irrigation system, drip irrigation without plastic mulch; FP, farmers’ practices system; BS, before sowing stage; V6, sixth leaf stage; VT, flowering stage; R6, harvest stage.

Based on the Bray-Curtis distance dissimilarity, PCoA exhibited more than half of the variation of the bacterial community composition could be explained by the first and second principal coordinates, with the PCo1 accounted for about 31.71%-53.00% of the total variance, and the PCo2 accounted for about 10.88%-29.36% (Fig. 6). The PCoA revealed that the best separation into three groups according to irrigation and fertigation systems. For example, DI was separated from FP and MF on PCoA1 at BS, MF was separated from FP and DI on PCoA2 (Fig. 6A); MF was separated from FP and DI on PCoA1 at V6, FP was separated from DI and MF on PCoA2 (Fig. 6B); FP was separated from DI and MF on PCoA1 at VT and R6, MF was separated from FP and DI on PCoA2 (Figs. 6C and 6D). Adonis analysis also showed significant community differences derived from different three agricultural irrigation and fertigation systems (Table 2).

**Table 2:** The effects of three agricultural cropping patterns on bacterial community structure tested by Adonis based on the Bray-Curtis distance.

**Factor**	**Sums of sqs**	**Mean sqs**	***F*. Model**	*R* ^2^	**Sig.** ^a^
All samples	0.825	0.075	2.484	0.532	0.001
FP^b^	0.289	0.096	2.834	0.515	0.001
DI	0.222	0.074	2.502	0.484	0.001
MF	0.140	0.047	1.722	0.392	0.001

**Notes.**

a The significance (Sig.) was examined by *F* tests based on sequential sums of squares (SS) from 999 permutations of the OTU level.

b MF, mulched fertigation system, drip irrigation under plastic mulch; DI, drip irrigation system, drip irrigation without plastic mulch; FP, farmers’ practices system.

The predominant taxa in response to the differences of bacterial composition among three agricultural irrigation and fertigation systems were further assessed using LEfSe analysis. At the genus level, *Luteimicrobium*, *Castellaniella* and *Cupriavidus* were enriched in FP at BS (Figs. S2A and S3A); *Paenibacillus*, *Starkeya* and *Sphingobacterium* were enriched in MF at BS. *Rubrobacter*, *Sphingobium*, *Aquicella*, *Rhizorhapis*, *Paenarthrobacter* and *Hydrogenispora* were enriched in MF at V6 (Figs. S2B and S3B); *Ohtaekwangia*, *Saccharopolyspora*, *Phaselicystis* and *Craurococcus* were enriched in DI at V6; *Aeromicrobium*, *Pedobacter*, *Gemmata* and *Schlesneria* were enriched in FP at V6. *Gemmatimonas*, *Pseudolabrys*, *Streptosporangium*, *Chthonomonas*, *Luteimonas* and *Salinispora* were enriched in MF at VT (Figs. S2C and S3C); *Nocardioides*, *Altererythrobacter*, *Microbacterium*, *Methylobacterium* and *Cnuella* etc. were enriched in DI at VT; *Nitrospira*, *Archangium* and *Arcticibacter* were enriched in FP at VT. *Dongia*, *Rhodoplanes*, *Mesorhizobium, Aquicella* and *Pseudolabrys* etc. were enriched in MF at R6 (Figs. S2D and S3D); *Parafrigoribacterium*, *Rudaibacter*, *Mucilaginibacter*, *Nitriliruptoria* and *Novosphingobium* etc. were enriched in DI at R6; *Nitrospira*, *Aeromicrobium*, *Reyranella*, *Nitrosospira* and *Nitrosomonas* were enriched in FP at R6.

### Relationships between bacterial communities and physicochemical properties

Pearson correlation analysis manifested that there was a significant correlation between soil bacterial communities and soil physicochemical properties (Fig. S4). The bacterial abundance had a significantly negative correlation with pH (*P* < 0.05), while Shannon had a significantly negative correlation with ${\mathrm{NH}}_{4}^{+}$-N (*P* < 0.01). PCoA1 was significantly positively correlated with ${\mathrm{NO}}_{3}^{-}$-N (*P* < 0.05).

**Figure 7:**
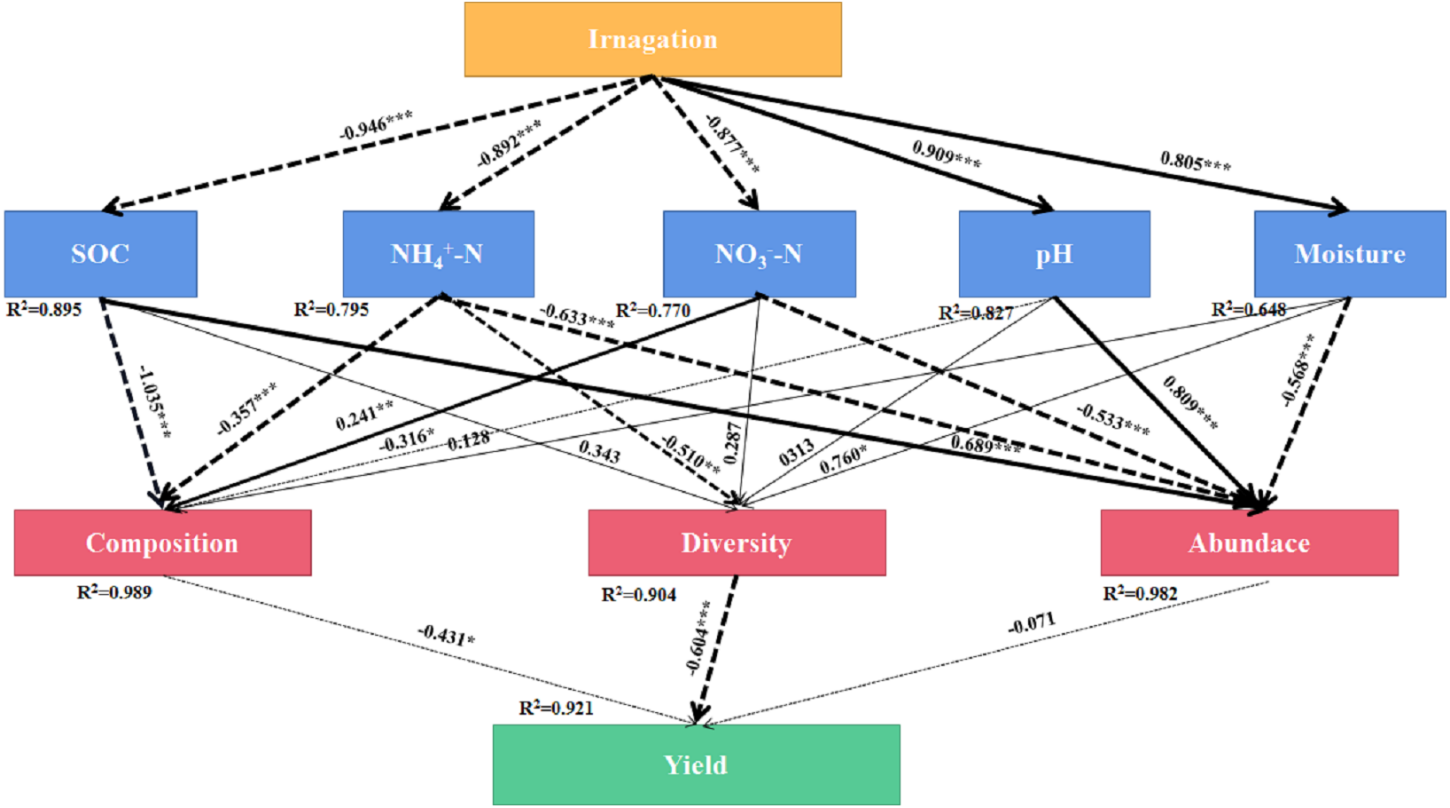
Final path with structural equation model (SEM) for the relationships among agricultural cropping patterns, soil chemical properties, bacterial traits and yields. Values above the line represent the path coefficients. Solid lines indicate positive path coefficients and dashed lines indicate negative path coefficients. The width of arrows indicates the strength of the standardized path coefficient. Asterisks (*, **, ***) indicate *P* < 0.05, *P* < 0.01 and *P* < 0.001, respectively. The data for the bacterial composition was calculated by principal coordinates analysis. The diversity was calculated by Shannon index. The abundance was calculated by soil bacterial 16S rRNA gene copy numbers. SOC, soil organic carbon; ${\mathrm{NH}}_{4}^{+}$-N, ammonium nitrogen; ${\mathrm{NO}}_{3}^{-}$-N, nitrate nitrogen; AN, available nitrogen; AP, available phosphorus; AK, available potassium.

SEM was used to evaluate the direct and indirect effects of irrigation on soil bacterial community composition, diversity, abundance, and yield (Fig. 7). The final equation model accord with the significance standard (*χ*^2^ = 91.65, *df* = 33, *P* = 0.001, RMSEA = 0.2, GFI = 0.63, AIC = 157). This model explained 92.1%, 98.9%, 90.4% and 98.2% of the variation in yield, bacterial community composition, diversity, and abundance, respectively. ${\mathrm{NO}}_{3}^{-}$-N showed direct effect on bacterial community composition (*λ* = 241, *P* < 0.01). Moisture showed direct effect on bacterial diversity (*λ* = 760, *P* < 0.05). SOC and pH showed direct effect on bacterial abundance (*λ* = 689, *P* < 0. 001, *λ* = 0.809, *P* < 0.001). The results showed that irrigation systems had indirect effects on community composition and bacterial diversity through SOC, ${\mathrm{NH}}_{4}^{+}$-N, ${\mathrm{NO}}_{3}^{-}$-N, pH, and moisture, however, ${\mathrm{NH}}_{4}^{+}$-N and ${\mathrm{NO}}_{3}^{-}$-N had indirect effects on yield through bacterial community composition, bacterial diversity and bacterial abundance.

### Co-occurrence patterns of three agricultural irrigation and fertigation systems

**Figure 8:**
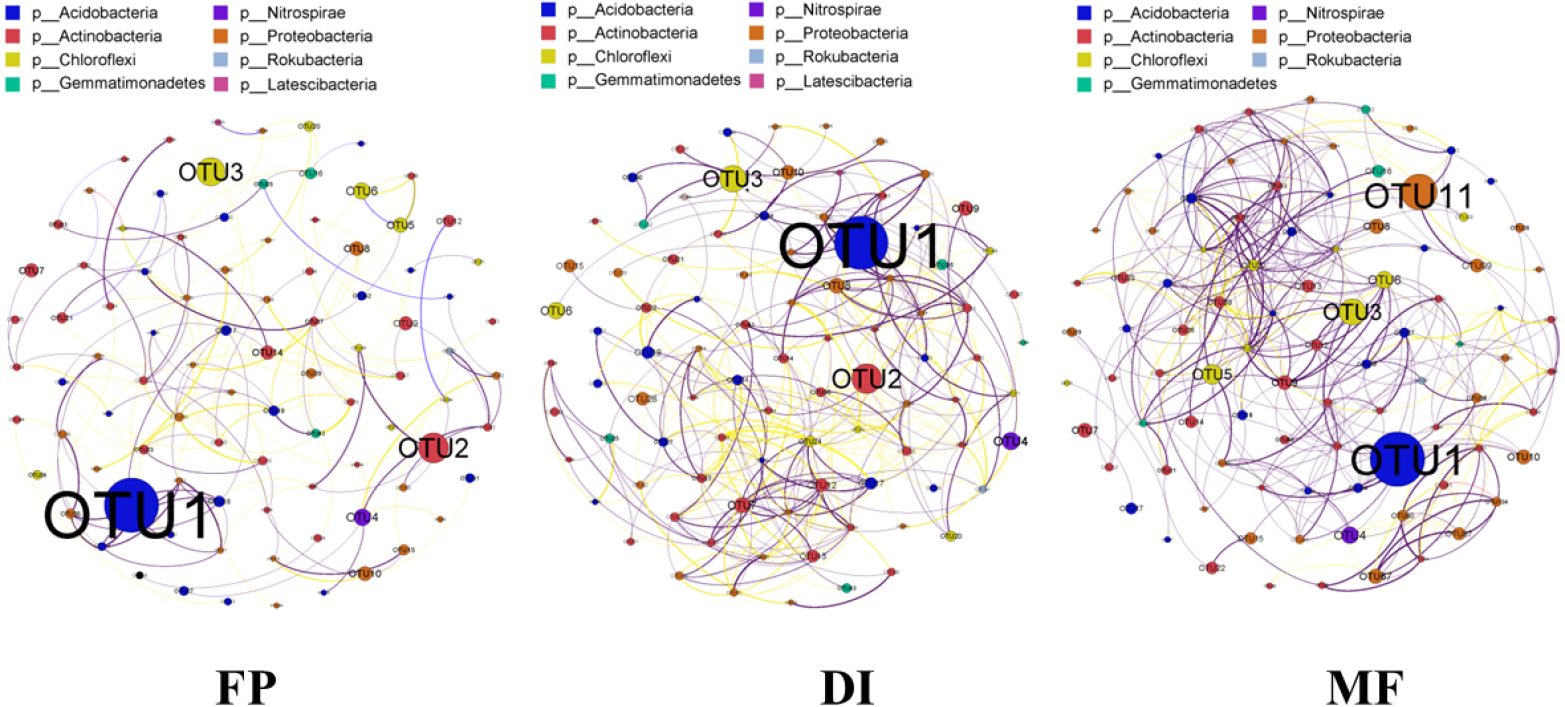
Bacterial co-occurrence networks of three agricultural cropping patterns from OTU profiles. A connection represented a strong (Spearman’s correlation coefficient *ρ* > 0.7) and significant (*P* < 0.05) correlation. The node colours in the network represent different major phyla, the node size represents its relative abundance, the connection thickness of the edge represents the value of Spearman’s correlation coefficients, a red line indicates a negative correlation between two individual nodes, and a green line indicates a positive correlation. MF, mulched fertigation system, drip irrigation under plastic mulch; DI, drip irrigation system, drip irrigation without plastic mulch; FP, farmers’ practices system.

The co-occurrence network was constructed based on the sequencing results of bacterial 16S rRNA gene in three agricultural irrigation and fertigation systems, and the top 100 OTUs of abundance were selected to construct the network. The interaction results in the community structure being affected by species. The co-occurrence network analysis revealed that the significantly different structure characteristics among the three agricultural irrigation and fertigation systems were from each other (Fig. 8). The co-occurrence network of FP, DI and MF consisted of 92, 90, 92 nodes and 158, 329, 359 edges, respectively. The nodes involved in co-occurrence network analysis mainly belonged to Acidobacteria, Actinobacteria, Chloroflexi and Gemmatimonadetes.

## Discussion

### Shifts in soil physicochemical properties under three agricultural irrigation and fertigation systems

In this study, soil moisture significantly increased in DI and MF compared with FP. This was because drip irrigation reduced the growth redundancy of roots and improved the root productivity of crop, thus achieving the goal of water-saving and increased yield (Wang et al., 2021). This was because especially the subsurface drip irrigation (in MF combined with plastic mulch) has strongly reduced evaporation and thus had led to the higher soil moisture. Our results showed that compared with FP, MF significantly decreased SOC, ${\mathrm{NH}}_{4}^{+}$-N and ${\mathrm{NO}}_{3}^{-}$-N contents. Li et al. (2020) also showed that compared with flood irrigation, drip irrigation significantly decreased SOC content. Tuo et al. (2010) showed that ${\mathrm{NH}}_{4}^{+}$-N content decreased and ${\mathrm{NO}}_{3}^{-}$-N content increased firstly and then decreased under the film. Thus, the decrease in ${\mathrm{NH}}_{4}^{+}$-N and ${\mathrm{NO}}_{3}^{-}$-N contents under MF might be due to crops on the uptake of nutrients, the decrease of SOC content mainly a result of an enhanced microbial decomposition due to higher soil temperature and moisture under the plastic mulch. Zhou & Feng (2020) also found that mulching enhanced soil water, and nutrient acquisition in shallow layer (0 −20 cm). In addition, maize yield in DI and MF were significantly higher (*P* < 0.05) than that of FP (Fig. 1). Bar-Yosef (1999) also manifested drip irrigation significantly improved both yield and nutrient use efficiency. Gao et al. (2019) indicated that optimal soil mulching to enhance maize yield with a meta-analysis. Wang et al. (2017b) indicated that flood irrigation and a single basal fertilizer application, had caused the high yield gap between what is achievable and farmers’ typical production, and low resource use efficiency.

### Soil bacterial abundance and community structure in response to three agricultural irrigation and fertigation systems

As expected, we demonstrated that MF significantly increased the bacterial abundance (Fig. 2). This was because as a local irrigation with small flow, drip irrigation caused the alternation of dry and wet soil, enhanced nutrient activation and promoted soil bacterial reproduction (Liu et al., 2014). Brockett, Prescott & Grayston (2012) also recognized that the soil moisture content was significantly positively related to microbial biomass. Drip irrigation had appropriate moisture retention and significantly increased the biomass of Gram-positive bacteria (Dangi et al., 2016). Under various irrigation systems, the amount of irrigation enhanced the bacterial abundance, and a humid environment was more conducive to bacteria reproduction. The alternation of dry and wet soil under drip irrigation systems enhanced nutrient activation (Muhr, Franke & Borken, 2010), and promoted the bacterial reproduction in the cultivated layer soil (Liu & Kang, 2014).

This study showed that the dominant bacterial phyla were Actinobacteria, Proteobacteria and Acidobacteria (Fig. 3), which was consistent with a previous study that also reported that the dominant phyla under farmland plastic film mulching were Proteobacteria, Acidobacteria, and Actinobacteria (Huang et al., 2019). DI and MF decreased the relative abundance of Acidobacteria compared with FP at R6 stage (Fig. S1). This may be due to the oligotrophic nature of Acidobacteria bacteria (Placella, Brodie & Firestone, 2012; Pascault et al., 2013). DI and MF led to an increase in the relative abundance of *Bradyrhizobium*, *Lysobacter* and *Rhodoplanes* (Fig. 4). These functional groups were associated with the nitrogen cycle (nitrogen fixation, nitrate denitrification and ureolysis). *Bradyrhizobium* was related to nitrogen fixation and nitrate denitrification, *Rhodoplanes* was related to nitrate denitrification, *Lysobacter* was related to ureolysis (Wang et al., 2018a). These findings illustrated that MF and DI promoted the reproduction of microorganisms, so as to improve soil nitrogen turnover in the nutrient cycle under the agro-ecosystem. DI and MF led to an increase in the relative abundance of *Rubrobacter* and *Streptomyces*. *Rubrobacter* is a functional bacteria related to carbon metabolism and improves the breakdown of xylan (Garcia et al., 2009). Xylan is a kind of heteropolysaccharide existing in the plant cell wall. It is a major component of plant hemicellulose and a complex five-carbon organic matter. *Streptomyces* could decompose chitin, a polysaccharide with the same structure, which is polymerized by N-acetylglucosamine through *β*-linkage. It is widely distributed in crustacean shells, insect crustaceans, fungal cell walls, and some green algae (Diwan, Mukhopadhyay & Gupta, 2020). These results implicated that MF and DI showed a significant increase in some beneficial bacterial species compared with FP.

In this study, DI and MF increased bacterial diversity and richness (Fig. 5). Fu et al. (2021) also manifested that drip irrigation improves bacterial diversity and richness. The decrease in soil moisture in arid regions significantly reduced the soil microorganism diversity and abundance (Maestre et al., 2015). This is because microorganisms will need to adapt to lower soil water potential during the drought, which through dehydration into a dormant state, or through the accumulation of solute to adapt to the drought environment. When drought occurs, microorganisms invest in the accumulation of solute and suffer from drought living conditions, and may lead to microbial dormancy or death, which affects its richness and diversity (Jabro et al., 2008; Muhr, Franke & Borken, 2010).

### Correlations between soil physicochemical properties and bacterial community structure under three agricultural irrigation and fertigation systems

The soil bacterial diversity changed under DI and MF, and these variations were closely related to soil properties. The soil bacterial alpha diversity was significant negatively correlated with AN and SOC (Fig. S4). The soil bacterial community composition was significantly negatively correlated with ${\mathrm{NH}}_{4}^{+}$-N and SOC (Fig. 7). Under a subsurface drip irrigation system, the growth and proliferation of other bacteria constricted the niche of the organic matter-metabolizing bacteria in the soil (Kaymak, 2011), resulting in a decline in their relative abundance, and the relative abundance of organic matter-metabolizing bacteria negatively correlated with the crop growth indicators to a certain degree (Wang et al., 2018b). Similar to this result, Huang et al. (2019) illustrated that the bacterial diversity had a negative correlation with TN and SOC, diversity mainly depended on TN, and soil moisture was significantly related to changes in the distribution of bacterial communities under farmland mulching. Li et al. (2014) also found that soil nutrient availability had an impact on soil bacterial diversity. These results can be explained by the competition between crops and soil microorganisms for mineral nutrients (Miatto, Wright & Batalha, 2016), the soil microbial activity will accelerate the decomposition of organic matter. Furthermore, crops need more soil nutrients during the vigorous period, hence a large amount of soil nitrogen and carbon are absorbed by crops (Li et al., 2004). The soil bacterial diversity was significantly positively correlated with soil moisture, and soil bacterial community composition was significantly positively correlated with soil pH (Fig. 7). Lauber et al. (2008) found that irrigation system would change the bacterial community structure, and the community composition was significantly related to soil water content and soil pH. Huang et al. (2019) showed that the changes of the bacterial community distribution under farmland mulching practices were significantly related to the soil moisture, which varied significantly with a range of 9.8–17.3 between treatments. Obayomi et al. (2020) indicated that there was a significant relation to soil pH with the soil bacterial richness and diversity under wastewater irrigation and plastic mulch. The higher correlation between soil microbial diversity and nutrient content, the more intense the competition between crops and soil microorganisms (Miki, 2012).

### The co-occurrence network under three agricultural irrigation and fertigation systems

The nodes and edges in MF were greater than those in FP and DI, indicating that the MF bacterial network was larger and the relationships among species were more complex (Fig. 8). The complex co-occurrence network may be the direct factor leading to the increase of diversity in MF. The MF network had more positive correlation connections, and OTUs had more cooperative relations, which could better buffer the interference brought by the external environment. The bacterial network in MF was more conducive to the health and stability of agroecosystem. The relationship between species in the co-occurrence network analysis can be described as positive correlation and negative correlation. Positive correlation indicated that the niche of the species was the same or had a symbiotic relationship, while negative correlation represented a competition or predation relationship (Layeghifard, Hwang & Guttman, 2016). The three networks in this study were all dominated by positive correlation, indicating that the bacteria in FP, DI and MF was mainly cooperative, and the competition was weak.

## Conclusions

In summary, mulched fertigation system significantly altered the soil properties and bacterial community composition, especially beneficial bacterial reproduction. Moreover, MF significantly enhanced the soil bacterial diversity and richness. The agricultural irrigation and fertigation systems had indirect effects on bacterial community composition and diversity through SOC, ${\mathrm{NH}}_{4}^{+}$-N, ${\mathrm{NO}}_{3}^{-}$-N, pH and moisture, and ${\mathrm{NH}}_{4}^{+}$-N and ${\mathrm{NO}}_{3}^{-}$-N had indirect effects on yield through bacterial community composition, diversity, and bacterial abundance. In addition, MF bacterial network was more conducive to the health and stability of agroecosystem since it was a good system to increase soil bacterial diversity and richness, and to maintain the functional stability of soil biological processes.

##  Supplemental Information

10.7717/peerj.14044/supp-1 Figure S1 Bacterial phyla with significant shifts in relative abundance in response to FP vs DI and FP vs MF at V6, VT and R6 (at 95% confidence intervals, Student’s *t*-test, equal variance) MF, mulched fertigation system, drip irrigation under plastic mulch; DI, drip irrigation system, drip irrigation without plastic mulch; FP, farmers’ practices system; V6, sixth leaf stage; VT, flowering stage; R6, harvest stage.

10.7717/peerj.14044/supp-2 Figure S2 Taxonomic cladogram produced from the LEfSe analysis The phylum, class, order, family, and genus levels are listed in order from inside to outside of the cladogram, and the labels for levels of the family and genus are abbreviated using a single letter. Green, red and blue showed taxa enriched in FP, DI and MF, respectively, while the yellow circles represented the taxa without significant differences among the three agricultural cropping patterns at BS (a), V6 (b), VT (c) and R6 (d). MF, mulched fertigation system, drip irrigation under plastic mulch; DI, drip irrigation system, drip irrigation without plastic mulch; FP, farmers’ practices system; BS, before sowing stage; V6, sixth leaf stage; VT, flowering stage; R6, harvest stage.

10.7717/peerj.14044/supp-3 Figure S3 Histogram of the linear discriminant analysis (LDA) scores among the three agricultural cropping patterns at BS (a), V6 (b), VT (c) and R6 (d) , *P*< 0.05; LDA score 2.0 MF, mulched fertigation system, drip irrigation under plastic mulch; DI, drip irrigation system, drip irrigation without plastic mulch; FP, farmers’ practices system; BS, before sowing stage; V6, sixth leaf stage; VT, flowering stage; R6, harvest stage.

10.7717/peerj.14044/supp-4Figure S4Pearson correlation coefficients between soil chemical properties and bacterial traits*, ** indicate *P* < 0.05, *P* < 0.01. MF, mulched fertigation system, drip irrigation under plastic mulch; DI, drip irrigation system, drip irrigation without plastic mulch; FP, farmers’ practices system; SOC, soil organic carbon; ${\mathrm{NH}}_{4}^{+}$-N, ammonium nitrogen; ${\mathrm{NO}}_{3}^{-}$-N, nitrate nitrogen; AN, available nitrogen; AP, available phosphorus; AK, available potassium.

10.7717/peerj.14044/supp-5 Table S1 Illumina MiSeq sequenced bacterial data (at 97% sequence similarity) based on the 16S rRNA gene

10.7717/peerj.14044/supp-6 Table S2 Maize yield in three agricultural cropping patterns(kg ha^−1^)

10.7717/peerj.14044/supp-7 Table S3 16S rRNA gene copy numbers × 108 g-1 dry soil

10.7717/peerj.14044/supp-8 Table S4 Taxonomic composition of bacterial communities at the phylum level under three agricultural cropping patterns

10.7717/peerj.14044/supp-9 Table S5 The relative abundance of dominant bacterial genera under three agricultural cropping patterns

10.7717/peerj.14044/supp-10 Table S6 Shannon index and Chao index of bacterial communities for three agricultural cropping patterns at BS, V6, VT and R6

10.7717/peerj.14044/supp-11 Table S7 Soil chemical properties under three agricultural cropping patterns

## Additional Information and Declarations

### Data Availability

The following information was supplied regarding data availability: The data is available at NCBI: SRP365802.
