# Supplementary figures and images for: Dynamics of soil properties and bacterial community structure by mulched fertigation system in semi-arid area of Northeast China
Source: PeerJ. 2022 Sep 22;10:e14044. doi: 10.7717/peerj.14044 (PMC9509672; doi:10.7717/peerj.14044)

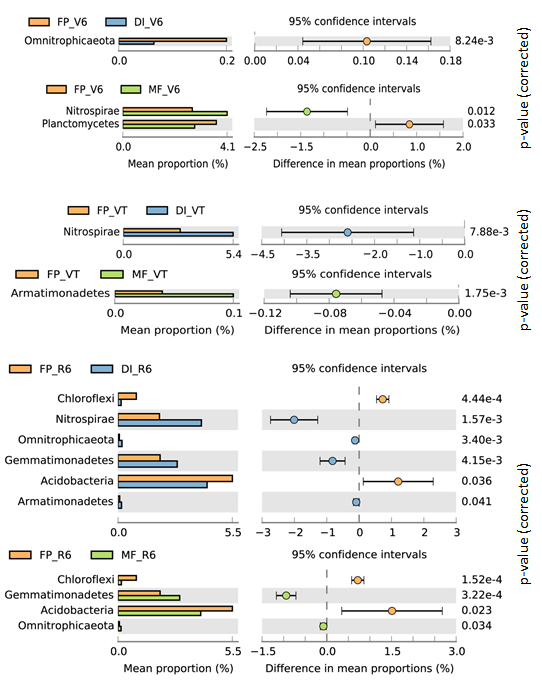

Supplement: Figure S1 — MF, mulched fertigation system, drip irrigation under plastic mulch; DI, drip irrigation system, drip irrigation without plastic mulch; FP, farmers’ practices system; V6, sixth leaf stage; VT, flowering stage; R6, harvest stage. [file peerj-10-14044-s001.png]

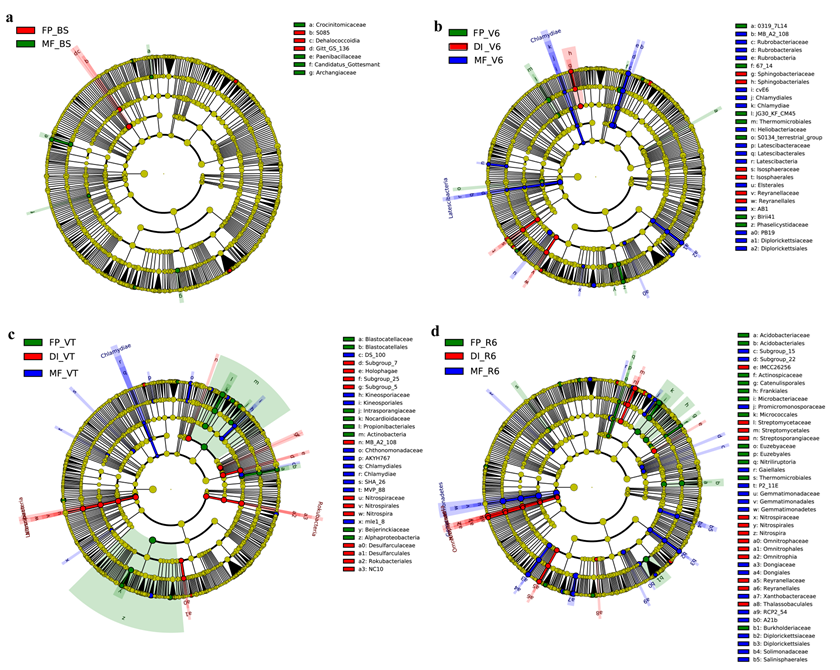

Supplement: Figure S2 — The phylum, class, order, family, and genus levels are listed in order from inside to outside of the cladogram, and the labels for levels of the family and genus are abbreviated using a single letter. Green, red and blue showed taxa enriched in FP, DI and MF, respectively, while the yellow circles represented the taxa without significant differences among the three agricultural cropping patterns at BS (a), V6 (b), VT (c) and R6 (d). MF, mulched fertigation system, drip irrigation under plastic mulch; DI, drip irrigation system, drip irrigation without plastic mulch; FP, farmers’ practices system; BS, before sowing stage; V6, sixth leaf stage; VT, flowering stage; R6, harvest stage. [file peerj-10-14044-s002.png]

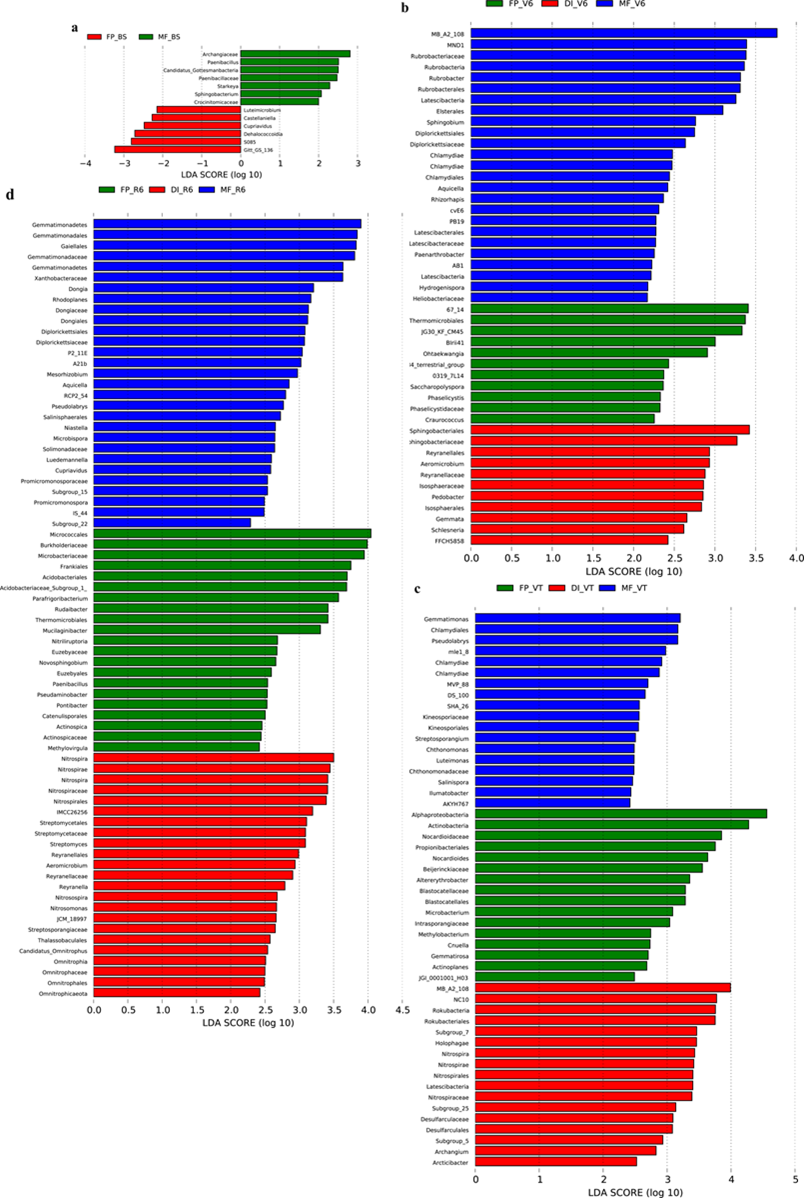

Supplement: Figure S3 — MF, mulched fertigation system, drip irrigation under plastic mulch; DI, drip irrigation system, drip irrigation without plastic mulch; FP, farmers’ practices system; BS, before sowing stage; V6, sixth leaf stage; VT, flowering stage; R6, harvest stage. [file peerj-10-14044-s003.png]

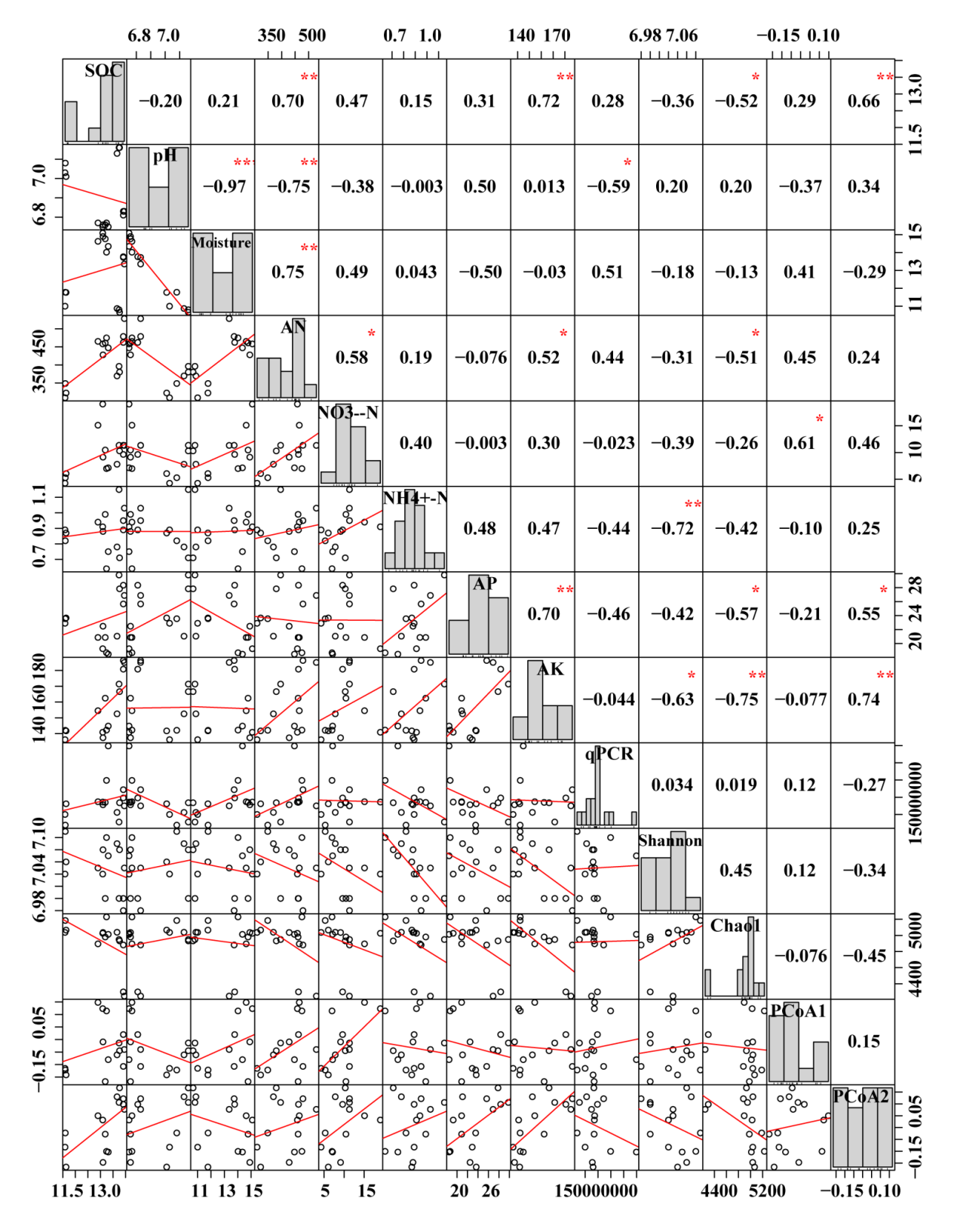

Supplement: Figure S4 — *, ** indicate P < 0.05, P < 0.01. MF, mulched fertigation system, drip irrigation under plastic mulch; DI, drip irrigation system, drip irrigation without plastic mulch; FP, farmers’ practices system; SOC, soil organic carbon; ${\mathrm{NH}}_{4}^{+}$NH4+-N, ammonium nitrogen; ${\mathrm{NO}}_{3}^{-}$NO3−-N, nitrate nitrogen; AN, available nitrogen; AP, available phosphorus; AK, available potassium. [file peerj-10-14044-s004.png]
