# Supplementary material for: Dynamics of soil properties and bacterial community structure by mulched fertigation system in semi-arid area of Northeast China
Source: PeerJ. 2022 Sep 22;10:e14044. doi: 10.7717/peerj.14044 (PMC9509672; doi:10.7717/peerj.14044)
Supplement: Table S1 [file peerj-10-14044-s005.docx]

| **Table S1** **Illumina MiSeq sequenced bacterial data (at 97% sequence similarity) based on the 16S rRNA gene.** | | | | |
| --- | --- | --- | --- | --- |
| **Sample** | **Sequences** | **Bases(bp)** | **Average Length (bp)** | **OTU** |
| DI_BS_1 | 33240 | 12529461 | 376.94 | 3682 |
| DI_BS_2 | 31839 | 11999413 | 376.88 | 3474 |
| DI_BS_3 | 36382 | 11253522 | 376.96 | 4181 |
| DI_BS_4 | 32478 | 12186754 | 376.92 | 3587 |
| DI_R6_1 | 32634 | 12300754 | 376.93 | 4208 |
| DI_R6_2 | 33768 | 12729099 | 376.96 | 3630 |
| DI_R6_3 | 30010 | 11312654 | 376.96 | 4099 |
| DI_R6_4 | 31874 | 12197496 | 379.93 | 4195 |
| DI_V6_1 | 39739 | 12517236 | 376.93 | 4020 |
| DI_V6_2 | 32804 | 12362977 | 376.87 | 4268 |
| DI_V6_3 | 35962 | 13553910 | 376.9 | 3910 |
| DI_V6_4 | 33652 | 12746397 | 376.91 | 4147 |
| DI_VT_1 | 45187 | 17037310 | 377.04 | 4319 |
| DI_VT_2 | 49383 | 22388360 | 377.02 | 3565 |
| DI_VT_3 | 44262 | 16684939 | 376.96 | 3849 |
| DI_VT_4 | 44728 | 16836254 | 377.01 | 3736 |
| FP_BS_1 | 42890 | 19935468 | 376.92 | 3678 |
| FP_BS_2 | 35842 | 13506479 | 376.83 | 4123 |
| FP_BS_3 | 42894 | 16169531 | 376.96 | 3799 |
| FP_BS_4 | 40372 | 15839462 | 376.93 | 3745 |
| FP_R6_1 | 44488 | 16999915 | 376.94 | 3414 |
| FP_R6_2 | 49985 | 18838131 | 376.88 | 3768 |
| FP_R6_3 | 46422 | 17469852 | 376.33 | 4041 |
| FP_R6_4 | 44372 | 17574832 | 376.87 | 3726 |
| FP_V6_1 | 33998 | 12816244 | 376.97 | 3749 |
| FP_V6_2 | 34164 | 13416943 | 376.95 | 4049 |
| FP_V6_3 | 35113 | 13235239 | 376.93 | 3761 |
| FP_V6_4 | 34982 | 13274985 | 376.93 | 3725 |
| FP_VT_1 | 46285 | 17409648 | 376.14 | 3191 |
| FP_VT_2 | 51944 | 19578409 | 376.91 | 3937 |
| FP_VT_3 | 44940 | 16942170 | 377 | 3750 |
| FP_VT_4 | 45837 | 17193746 | 376.93 | 3736 |
| MF_BS_1 | 43659 | 16454798 | 376.89 | 4180 |
| MF_BS_2 | 44868 | 20680514 | 376.91 | 3622 |
| MF_BS_3 | 45239 | 17046896 | 376.82 | 3789 |
| MF_BS_4 | 43726 | 18746246 | 376.82 | 3632 |
| MF_R6_1 | 49451 | 18642737 | 376.99 | 3561 |
| MF_R6_2 | 46002 | 13572300 | 376.99 | 3650 |
| MF_R6_3 | 44590 | 16807598 | 376.94 | 3459 |
| MF_R6_4 | 46273 | 16492874 | 376.99 | 3487 |
| MF_V6_1 | 30379 | 11449727 | 376.9 | 3332 |
| MF_V6_2 | 35167 | 13256420 | 376.96 | 4166 |
| MF_V6_3 | 41420 | 15613237 | 376.95 | 4144 |
| MF_V6_4 | 36819 | 13927462 | 376.95 | 4028 |
| MF_VT_1 | 46874 | 17442308 | 377.01 | 3943 |
| MF_VT_2 | 39091 | 14737822 | 377.01 | 3818 |
| MF_VT_3 | 41922 | 15803442 | 376.97 | 4016 |
| MF_VT_4 | 41847 | 15304972 | 377.01 | 3846 |
