# Supplementary material for: Dynamics of soil properties and bacterial community structure by mulched fertigation system in semi-arid area of Northeast China
Source: PeerJ. 2022 Sep 22;10:e14044. doi: 10.7717/peerj.14044 (PMC9509672; doi:10.7717/peerj.14044)
Supplement: Table S2 [file peerj-10-14044-s006.docx]

| **Table S2 Maize yield in three agricultural cropping patterns(kg ha^-1^)** | | | | |
| --- | --- | --- | --- | --- |
| **FP** | 10040.308 | 10607.169 | 10116.513 | 10307.997 |
| **MF** | 12085.792 | 12335.191 | 12420.291 | 12293.758 |
| **DI** | 11585.792 | 12135.191 | 12130.291 | 11817.092 |
